# Genome-wide profiling of G protein-coupled receptors in cerebellar granule neurons using high-throughput, real-time PCR

**DOI:** 10.1186/1471-2164-12-241

**Published:** 2011-05-16

**Authors:** Benjamin Maurel, Anne Le Digarcher, Christelle Dantec, Laurent Journot

**Affiliations:** 1 Institut de Genomique Fonctionnelle, 141 rue de la cardonille, F-34094 Montpellier Cedex 05, France; 2 CNRS, UMR5203, Montpellier, France; 3 Inserm, U661, Montpellier, France; 4 Univ Montpellier, Montpellier, France; 5 Montpellier GenomiX, Montpellier, France

## Abstract

**Background:**

G protein-coupled receptors (GPCRs) are major players in cell communication, regulate a whole range of physiological functions during development and throughout adult life, are affected in numerous pathological situations, and constitute so far the largest class of drugable targets for human diseases. The corresponding genes are usually expressed at low levels, making accurate, genome-wide quantification of their expression levels a challenging task using microarrays.

**Results:**

We first draw an inventory of all endo-GPCRs encoded in the murine genome. To profile GPCRs genome-wide accurately, sensitively, comprehensively, and cost-effectively, we designed and validated a collection of primers that we used in quantitative RT-PCR experiments. We experimentally validated a statistical approach to analyze genome-wide, real-time PCR data. To illustrate the usefulness of this approach, we determined the repertoire of GPCRs expressed in cerebellar granule neurons and neuroblasts during postnatal development.

**Conclusions:**

We identified tens of GPCRs that were not detected previously in this cell type; these GPCRs represent novel candidate players in the development and survival of cerebellar granule neurons. The sequences of primers used in this study are freely available to those interested in quantifying GPCR expression comprehensively.

## Background

GPCRs are seven transmembrane-domain receptors that are selectively activated by a wide array of ligands such as hormones, neurotransmitters, metabolites, odorants, pheromones, gustatory molecules and photons. They constitute as much as 3-6% of the genes encoded in the mammalian genomes [1]. Endo-GPCRs, *i.e*. those GPCRs that have a demonstrated or putative endogenous ligand, represent about one fourth of all GPCRs; the remaining ones are activated by odorants, pheromones, gustatory molecules and light. From a functional point of view, GPCRs have a role in virtually every physiological function [2]. It is therefore not surprising that approximately 45% of clinically relevant molecules target a GPCR [3]. Furthermore, ~35% of all endo-GPCRs are orphans, *i.e*. they have no identified ligand, and constitute an attractive reservoir of pharmacological targets.

It is of major interest to many academic and pharma/biotech scientists to determine the repertoire of GPCRs expressed in a given tissue or cell type, for instance to identify novel pharmacological targets in a murine model of a human disease. Furthermore, the remodeling of the GPCR repertoire during the development of the pathology in such a model may help prioritize which GPCRs should be targeted first.

Most cells express a small number of a given GPCR protein at their surface. Accordingly, these genes are usually expressed at low levels [4], which makes their accurate quantification by microarray-based detection systems challenging. RNA deep sequencing has the power to accurately determine expression levels of weakly expressed genes, at the expense of costs, however. Real-time RT-PCR is reputedly more sensitive than microarrays, but its throughput was limited until recently. Real-time PCR machines in the 384-, 1,536-, 3,200- or 9,216-well format became recently available and affordable. This prompted us to develop a collection of primers that can be used in real time RT-PCR experiments to characterize GPCR expression genome-wide. We first draw an inventory of all endo-GPCRs encoded in the murine genome. We then designed and validated a collection of primers for quantitative RT-PCR experiments, and selected adequate statistical tests. To illustrate the usefulness of this approach, we determined the repertoire of GPCRs expressed in cerebellar granule neurons and neuroblasts during postnatal development. We identified tens of GPCRs that were not detected previously in this cell type; these GPCRs represent novel candidate players in the development and survival of cerebellar granule neurons.

## Results and discussion

### Identification of endo-GPCRs

Our first aim was to identify endo-GPCRs encoded by the murine genome as comprehensively as possible. We originally started from Unigene clusters annotated as "receptor", and manually sorted known GPCRs and seven transmembrane- (7TM) proteins. At that step, we excluded 7TM receptors related to odorant and pheromone receptors. To discover genes encoding novel 7TM receptors, we blasted the murine genome with the transmembrane domain of the 263 7TM proteins selected from Unigene. The process was reiterated with novel sequences not annotated as GPCR in the original Unigene set until no novel sequence could be discovered. The transcription of the sequences identified in this way was confirmed by searching the murine EST database. This strategy allowed us to identify 382 murine endo-GPCRs, which are listed in Additional file 1. The number of GPCR we identified is in line with previous studies that used bioinformatics and/or literature mining [4-8].

A special point should be made regarding the murine Mas-related family of GPCR (*Mrgpr*). As shown previously [9,10], the murine Mrg family consists of six single-copy genes (*Mrgprd*, *Mrgpre*, *Mrgprf*, *Mrgprg*, *Mrgprh*/*Gpr90*, and *Mas1*), as well as three large subfamilies (*Mrgpra*, *Mrgprb*, and *Mrgprc*) that together comprise about 50 distinct sequences. Members of the *Mrgpra *subfamily are poorly characterized, and many of them are predicted sequences or gene models that have no or very few experimental supporting data. The *Mrgprc *family members are all pseudo-genes that do not encode GPCRs [9]. As far as expression patterns are concerned, *Mrgprd *and the members of the *Mrgpra *family are expressed exclusively in the sensory neurons and in the trigeminal ganglia [9]. In contrast, *Mrgprb1-5*, *Mas1*, *Mrgpre*, *Mrgprf*, *Mrgprg*, and *Mrgprh*/*Gpr90 *are not expressed in dorsal root ganglia [9]. We therefore decided to focus on members of the *Mrgpra *subfamily that have been sufficiently characterized (*Mgrpra1-4*, *Mrgpra6*), members of the *Mrgprb *subfamily (*Mrgprb1-5*, *Mrgprb10*), and the six single-copy *Mrgpr*. Because ambiguity could not be resolved, we did not include in the GPCR collection 9 sequences with homologies to the *Mrgpra *family and 4 sequences with homologies to the *Mrgprb *family. It is therefore likely that additional members of the Mas-related family of GPCR do exist.

In addition to GPCRs, we also included in our analysis 7 genes encoding proteins that are not GPCR but rather alter GPCR pharmacology and/or coupling specificity. The *Ramp1-3 *(Receptor activity modifying protein) genes encode single transmembrane proteins that physically interact with receptors for calcitonin (Calcr) and calcitonin gene-related peptide (Calcrl). These 2 GPCRs are activated by a range of peptides, namely calcitonin, amylin, calcitonin gene-related peptide, adrenomedullin and intermedin/adrenomedullin 2, depending on the Ramp with which they interact [11]. In addition, efficient signal transduction by Calcr and Calcrl requires interaction with Crcp [12]. Knowledge about *Ramp1-3 *and *Crcp *expression is mandatory to interpret data about *Calcr *and *Calcrl *expression. These 4 genes were then included in the primer collection and annotated as 'GPCR-modifiers'. For similar reasons, we designed primers aimed at quantifying transcripts encoding the 3 secreted frizzled proteins, *i.e. Sfrp1*, *Sfrp2 *and *Sfrp3*/*Frzb *[13]. These genes are also included in Additional file 1.

### Design and validation of a primer collection for real-time PCR

Primers aimed at quantifying GPCR transcripts are available. One collection is far from comprehensive however, as it targets 274 GPCRs only [14]. The other commercially available collections are not comprehensive and not experimentally validated [8,15]. Furthermore, the commercial collections rely on Taqman^® ^probes, which ensure high specificity, but display lower sensitivity than SybrGreen^®^-based detection systems (see Additional file 2 for a comparison of 8 randomly selected Taqman assays to the corresponding primers used in this study). To be as comprehensive, sensitive, and reliable as possible, we designed and validated primer pairs suitable for quantification of every GPCR we identified using SybrGreen^®^-based detection systems. We focused exclusively on coding sequences, as the identification of the open reading frame (ORF) of a previously uncharacterized GPCR transcript is greatly facilitated by the characteristic 7TM topology. We therefore felt more confident in targeting the ORF region rather than the untranslated regions, specially for gene models and transcripts reconstructed from genomic sequences.

The validation of a primer pair requires the assessment of the specificity and efficiency of the amplification process, which in turn is possible only if the corresponding matrix is available. Because a number of the target GPCRs has never been studied previously, no corresponding sequence cloned in a plasmid is available; nor is available information about the pattern of the GPCR expression, which precluded the use of cDNAs. Hence we favored primers located in the same exon to allow the validation of the primer pairs using genomic DNA rather than cDNA or plasmids. This strategy is possible only if the cDNA mix that is to be used in real experiments is completely devoid of genomic DNA, which was routinely achieved by treatment of the RNA samples with RNase-free Dnase (data not shown). To validate each primer pair we performed real-time PCR with 6 different amounts of genomic DNA from100 pg (~50 copies of an autosomal gene) to 20 ng (~10,000 copies). We plotted the threshold cycle (Ct) *vs*. the log of the amount of input genomic DNA, and measured the slope and the correlation coefficient (r^2^) of the resulting linear regression straight line, and the Ct for 1 ng of input genomic DNA. We considered that a primer pair is acceptable if, in 2 independent tests, the slope was above -3.6 (>90% amplification efficiency), the correlation coefficient was above 0.95 and the Ct(1 ng) was below 30.

The specificity of the amplification product was verified in each experiment by inspection of the amplicon melting curve, which is expected to display a single peak at a temperature close to the theoretical amplicon melting temperature. Approximately 10% of the amplicons were loaded on an agarose gel to verify their size, and were sequenced on both strands. The sequences of the primers used in this study are available in the Additional file 3.

### Statistical analysis and normalization of PCR data

The next step was the selection of statistical tests suitable for testing differences in the expression levels of genes across the samples to be analyzed. The large scale, real-time PCR studies reported above either did not perform statistics or used parametric tests to test for differences in transcript abundance among samples. None of these studies corrected for multiple testing, which is obviously required given the large number of statistical tests to be performed in a single experiment. Parametric tests are more efficient than non-parametric tests in detecting differences between groups of values, but the distribution of the random variable should be known *a priori*. Student's t-test for instance assumes a Gaussian distribution. Similarly, although ANOVA does not call for normality, it is sensitive to heteroscedasticity. It was therefore important to determine if the PCR data can be assumed to be normally distributed, and whether the variance is dependent on initial target abundance or not. We randomly selected 9 primer pairs with GC-content ranging from 46 to 60% (Additional file 4) from the GPCR collection, and performed 64 replicate PCR on 50 pg, 500 pg, and 5 ng of genomic DNA. We plotted the distribution of the Ct values (Additional files 5, 6, 7, 8, 9, 10, 11, 12 and 13), and tested the normality of the distribution using a whole range of statistical tests (Additional file 4). Surprisingly, out of 9 primer pairs tested with 3 amounts of input genomic DNA, 55% produced a bimodal or a non-Gaussian distribution in at least one condition. Furthermore, 2/3 of the primers tested displayed a standard deviation that increased with decreased amount of input genomic DNA (Additional file 4), indicating heteroscedasticity. We concluded that we cannot assume the distribution of PCR data to be normal and homoscedastic, and we cannot routinely use parametric tests with high throughput PCR data. Similar experiments performed on 1 and 4 ng of cerebellar cDNAs led to the same conclusion (data not shown). Accordingly, we analyzed the PCR data using the Wilcoxon signed-rank and Kruskal-Wallis tests with Benjamini-Hochberg correction for multiple testing [16] as implemented in the non parametric module of the MeV 4.2 package [17]. For Kruskal-Wallis test, post-hoc tests were performed with the XLStat software (AddinSoft).

For real experiments, 2 rounds of normalization were routinely performed. To eliminate the plate-to-plate PCR variability, the data were normalized using the geometric average of 'house keeping' genes expression levels in a way similar to the one proposed by Vandesompele and co-workers [18]. However, in contrast to the setting by Vandesompele and co-workers, each PCR plate comprised a single cDNA sample and primer pairs for multiple genes, including the selected reference genes. The level of expression of each GPCR "X" was normalized to the geometric mean of the expression levels of the selected reference genes, R1 to R3, in the same PCR plate according to the formula:

The MeV 4.2 package [17] was used for further processing and analysis of the data. To reduce the variability introduced by reverse transcription (RT), the normalized, log2-transformed PCR data obtained from individual RT were mean-centered and scaled in SD units.

### GPCR regulation during apoptosis of cerebellar granule cells

In the nervous system, GPCRs are critically involved during the embryonic development. For instance, Sonic Hedgehog (Shh) and Frizzled (Fz) are critical players in brain patterning by controlling the activity of several GPCRs, *i.e*. Smoothened (Smo) and Wnt family members, respectively. In the adult brain, GPCRs are key modulators of synaptic activity. Accordingly, they are targeted by most psycho-active molecules.

To illustrate the usefulness of the high throughput PCR approach, we and others performed tissue profiling, which gives a static view of the GPCR repertoire in a mixture of cell types [8,19]. In the present work, we focused on a more dynamic setting, and monitored the remodeling of the GPCR repertoire in a single neuronal subtype. We used a cellular model of neuronal development that was previously studied in the laboratory [20,21], and which is highly appropriate for population-based studies using techniques such as microarrays and PCR. Cerebellar granule neurons (CGNs) constitute the most abundant neuronal population in the mammalian central nervous system, and can be cultured *in vitro *up to 98% homogeneity. Cerebella were collected at P7, which corresponds to the very ending of the granule neuroblast expansion period *in vivo*. The neuroblasts complete their last division approximately 1 day after plating, and exit the cell cycle to enter differentiation in a synchronous process. After 7 days in culture, the CGNs are fully differentiated and functional. The whole culture process takes place in the presence of depolarizing concentrations of potassium chloride ([KCl] = 25 mM; K25) that induce a phasic electrical activity. Depolarization is presumed to mimic the endogenous excitatory activity that is required for CGN survival during cerebellar development *in vivo *(Ikonomidou et al., 1999). Lowering [KCl] to 5 mM (K5) in the absence of serum triggers apoptosis [22]. This mimics the naturally occurring death that takes place in the external granular layer of newborn rodent cerebellum [23]. This apoptosis process is fully dependent on new RNA and proteins synthesis [22], and we previously identified genes regulated early after CGN apoptosis induction using microarrays [20]. Although a role for GPCR activation in preventing potassium withdrawal-induced apoptosis of CGNs is abundantly documented, we observed the regulation of a very limited number of genes encoding GPCRs in our previous microarray-based study. One reason might be that the regulation of GPCR activity during the apoptotic process does not involve gene regulation. An alternative explanation is the reputed lack of sensitivity of microarrays, which prompted us to use the very same samples as those used in our previous study [20] to monitor GPCR expression in K25- vs. K5-treated CGN cultures.

**Figure 1:**
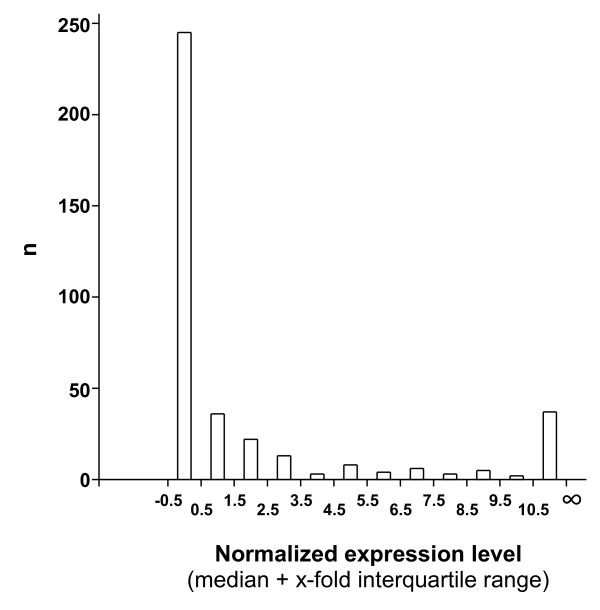
**Distribution of the abundance of transcripts encoding G protein-coupled receptors in differentiated cerebellar granule neurons**. Transcripts encoding 382 GPCRs were quantified in cerebellar granule neurons grown *in vitro *for 7 days (DIV 7) using real-time PCR. The distribution of transcript abundance was plotted with intervals equal to the interquartile range (IQR; the difference between the first and third quartile). The majority (298) of GPCRs are expressed at very low levels, *i.e*. between median - 0.5 IQR and median + 3.5 IQR. A population of 46 GPCRs displays intermediate expression levels (median + 4.5 IQR to median + 9.5 IQR), and 38 GPCRs are expressed above median + 10 IQR. The medium to high population of GPCRs likely corresponds to the physiologically most relevant GPCRs.

**Figure 2:**
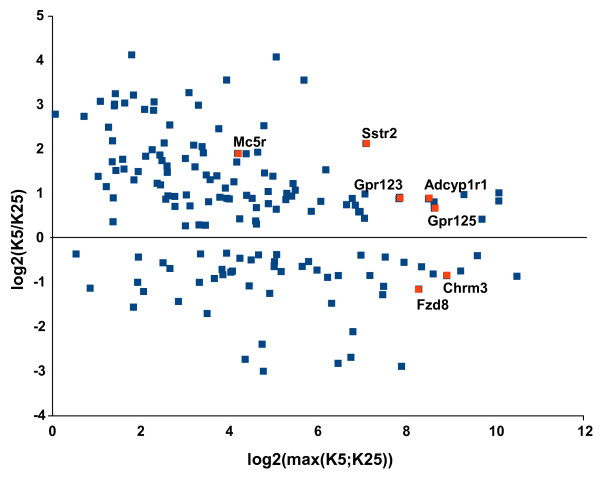
**MA plot of the expression levels of G protein-coupled receptors differentially expressed between healthy and early apoptotic cerebellar granule neurons**. Mouse cerebellar granule neurons grown *in vitro *for 7 days (DIV 7) were incubated for 4 hours in culture medium containing 25 mM (K25) or 5 mM (K5) KCl without serum. GPCR expression levels were determined using real-time PCR, and differentially expressed GPCRs were identified using the Wilcoxon signed-rank test with Benjamini-Hochberg correction. M is the log2-transformed ratio of the normalized expression levels of each GPCR in K5 and K25. A is the log2-transformed maximum of the normalized expression levels of each GPCR in K5 and K25. Red dots indicate those GPCRs that were also identified as differentially expressed in our previous microarray study [20] using the very same RNA samples. The real-time PCR approach identified more DE GPCRs than microarrays, especially among GPCRs expressed at low to medium levels.

We first draw the distribution of GPCR expression levels in K25 culture (Figure 1). We observed 3 populations of GPCRs. The vast majority of GPCRs (298) were expressed at very low levels (< median + 3.5 interquartile (IQR) range). In contrast, 38 GPCRs were abundantly expressed, *i.e*. >median + 10.5 IQR. A third population of GPCRs (46) displayed intermediate expression levels. With the notable exception of *Mc5r *that belongs to this intermediate population, those GPCRs that were shown to be differentially expressed (DE) using microarrays [20], *i.e. Sstr2*, *Chrm3*, *Gpr125*, *Gpr123*, *Fzd8 *and *Adcyp1r1*, were all among the most abundantly expressed GPCRs. These genes were also found as DE genes using the Wilcoxon signed-rank test with Benjamini-Hochberg correction on our qPCR data (Figure 2). In addition, we identified tens of DE GPCRs that were not identified using microarrays (Additional file 14). As shown on the MA plot displayed on Figure 2, most of the DE GPCRs with large fold-changes correspond to weakly expressed GPCRs. In contrast those DE GPCRs that were abundantly expressed displayed lower fold-changes. This confirms that the qPCR approach is more sensitive and has a larger dynamic range in fold-change than the microarray-based approach.

Interestingly, numerous GPCRs whose activation were previously shown to result in anti-apoptotic activity in this model, e.g. *Adcyap1r1 *[24], *Gabbr2 *[25]..., were found DE using the qPCR approach. As the CGNs were collected as early as 4 hours after potassium withdrawal, *i.e*. far before the first biochemical and morphological stigmata of apoptosis were discernible, it is likely that CGNs attempt to counteract the pro-apoptotic insult in part by up-regulating GPCRs whose activity is potentially beneficial, provided the cognate ligand is present in the extracellular milieu.

### GPCR regulation during post-natal development of cerebellar granule cells

Because of its stereotyped organization, the development of the cerebellum has been extensively studied [26]. The migration, proliferation, survival and differentiation of mouse cerebellar granule cells is controlled by a complex array of transcription factors and signaling pathways [27]. Direct GPCR ligands and modulators of GPCR activity, *e.g*. Shh, the Wnt proteins and Cxcl12/Sdf1, were shown to control different aspects of cerebellar granule differentiation, survival and positioning. However, the role of the majority of GPCRs expressed by cerebellar granule neuroblasts and neurons remains largely undefined.

We used the same *in vitro *culture model to monitor the repertoire of GPCRs expressed by cerebellar neuroblasts and during the postnatal differentiation and maturation of CGNs. Additional file 15 shows those GPCRs that were detectable in at least one condition, *i.e*. displayed Ct below 30 in at least 3 out of 15 PCR reactions. Not surprisingly, we found a number of GPCRs that were previously shown to influence various aspects of the CGN developmental process such as proliferation, migration, cell adhesion, survival and maturation. *Smoothened *(*Smo*) encodes a GPCR that interacts with the Shh receptor Ptch1, and mediates the proliferative action of Shh on neuroblasts [28]. *Smo *was highly expressed at day 0 when the proliferating neuroblasts were collected, and its expression decreased when the neuroblasts exited the cell cycle from day 1 on. Similarly, the most abundantly expressed GPCR at culture inception was *Gpr56*, that encodes an orphan GPCR controlling neuroblast adhesion [29]. *Cxcr4 *encodes the receptor for Cxcl12/Sdf1 (Stromal cell derived factor 1), and was shown to be essential for neuronal cell migration and patterning in the cerebellum [30]. We also found high expression of those GPCRs that are markers of mature CGNs because they are involved in the control of the electrical activity of these neurons, *i.e*. metabotropic GABA and Glutamate receptors. In addition, we identified tens of GPCRs that were not previously shown to be expressed in CGNs (Additional file 15) and are candidate players in the development and survival of CGNs.

**Figure 3:**
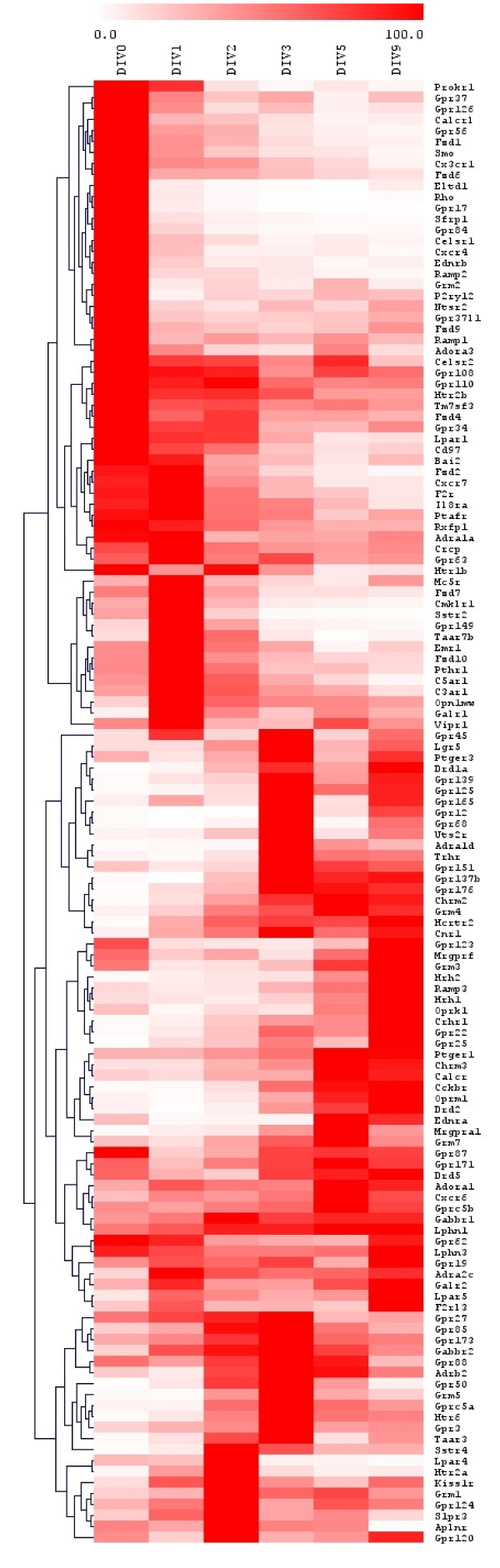
**Hierarchical clustering of G protein-coupled receptors differentially expressed during the development of cerebellar granule neuroblasts *in vitro***. Transcripts encoding 382 GPCRs were quantified using real-time PCR throughout the developmental process that leads from cerebellar granule neuroblasts to mature neurons *in vitro*. Data represent the average of 3 independent reverse transcription and PCR experiments. To take into account technical variations, the level of expression of each GPCR was normalized to the geometric mean of the expression levels of 3 selected reference genes, *i.e. B2m*, *Gapdh*, and *Gusb*. A Kruskal-Wallis test with Benjamini-Hochberg correction identified those GPCRs differentially expressed (DE) at one time point at least. Each GPCR was then expressed as the percentage of the maximal expression levels of that GPCR during the time course. DE GPCRs were classified according to their expression pattern using a hierarchical clustering algorithm as implemented in MeV 4.2.

Among expressed GPCRs, we sought to identify those that were regulated during the developmental time course as this may be an indication of a specific role for these GPCRs during CGN differentiation A Kruskal-Wallis test with Benjamini-Hochberg correction identified those GPCRs differentially expressed in at least one condition. DE GPCRs classification using a hierarchical clustering algorithm [17] revealed that a large number of GPCRs are very dynamically regulated during CGN development (Figure 3), suggesting that CG neuroblasts and mature neurons are sensitive to, at least in part, different sets of extracellular cues.

**Figure 4:**
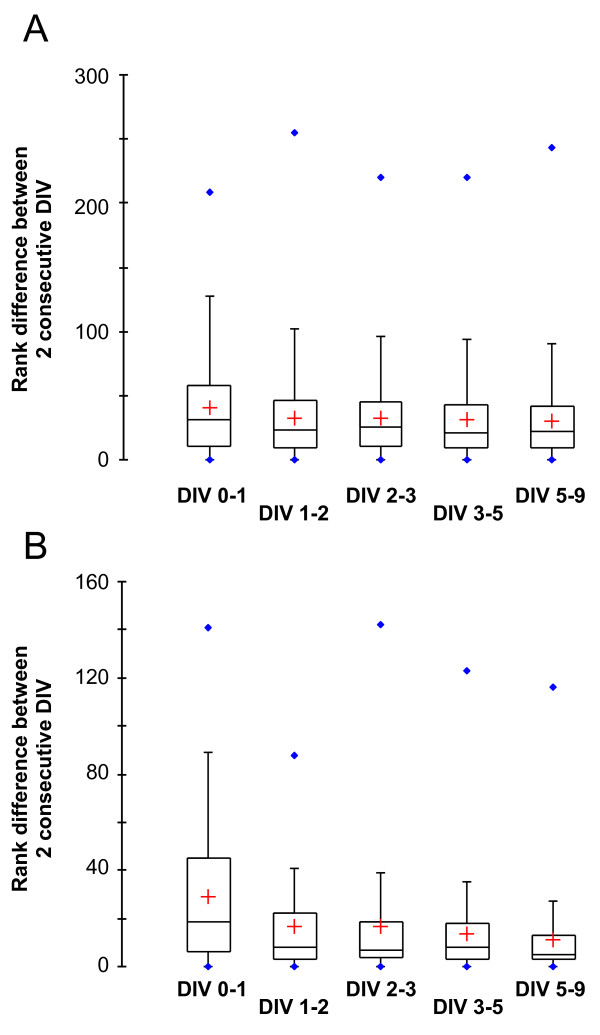
**The repertoire of GPCRs expressed by mature cerebellar granule neurons is mostly determinate after 3 days *in vitro *(DIV 3)**. To monitor global changes in GPCR expression, GPCRs were ranked by their expression levels, and the absolute difference in the ranks of each GPCR between 2 consecutive time points, *i.e*. DIV 0-1, DIV 1-2, DIV 2-3 and so on, were recorded. Box plots were drawn to visualize the distributions of the differences in GPCR expression levels between 2 consecutive DIV. Box plots are shown for both the whole GPCR set (Figure 4A) and for the 50 most abundantly expressed GPCRs at DIV 9 (Figure 4B). Whereas the distributions are comparable for the whole GPCR set, the box plots for the 50 most expressed GPCRs display a decrease in the average difference between 2 consecutive DIV before and after DIV 3, indicating that the repertoire of GPCRs expressed by mature cerebellar granule neurons is mostly determinate at DIV 3, when neurons are not morphologically mature yet.

We then sought to determine if the remodeling of the GPCR repertoire is continuous throughout the developmental process or whether there is a specific period at which remodeling is more active, and the GPCR repertoire becomes fixed. We first ranked the GPCRs according to their expression levels at each time point. We then calculated for each GPCR the absolute rank difference between 2 consecutive time points. The more remodeling of the GPCR repertoire, the higher the average rank difference between 2 consecutive time points. We then compared the distribution of the rank difference for the whole GPCR set (Figure 4A) and for the 50 most abundantly expressed GPCRs at DIV 9 (Figure 4B). For the whole GPCR set, the average rank variation is similar at each time point (Figure 4A). This is due to the fact that the ranks of the majority of weakly expressed GPCRs vary more or less randomly as their expression is biologically not significant. In contrast, when only the 50 most abundantly expressed GPCRs are considered, the distributions display a decrease in the average difference between 2 consecutive time points, before and after DIV 3 (Figure 4B). This indicates that the repertoire of GPCRs expressed by mature, DIV9 CGNs is largely determinate at DIV3. Interestingly, DIV3 CGNs do not display morphological and functional features of mature CGNs such as extended neurites, synaptic connections and electrical activity. Hence, the remodeling of the GPCR repertoire precedes neurite outgrowth, establishment of synaptic connections or electrical activity. Similarly, it does not depend on environmental and positional cues, which are obviously disrupted or abolished in the *in vitro *culture system. We concluded that the remodeling of the GPCR repertoire that occurs during postnatal CGN development is a robust, inbuilt process characteristic of CG neuroblasts exiting the cell cycle.

## Conclusions

This work identified the complete repertoire of GPCR encoded by the murine genome. Based on this information, we designed and validated a collection of primer pairs that allows the specific and sensitive quantification of GPCR transcripts using real-time PCR. We showed that non parametric statistical tests should be used routinely to identify variations of GPCR transcript abundance. Finally, we used this tool to characterize the repertoire of GPCRs expressed in cerebellar granule neurons, the most abundant central nervous system neuronal population. We showed that remodeling of the GPCR repertoire is an early event following cell cycle withdrawal of cerebellar neuroblasts, which is independent of environmental cues, neurite outgrowth and synaptic connections. This work paves the way towards the systematic identification of GPCRs expressed and regulated in murine models of human pathologies, offering the potential to identify novel, drugable pharmacological targets for these pathologies.

## Methods

### *in vitro *culture of mouse cerebellar granule neuroblasts

Design of the animal research was approved by the Institut de Génomique Fonctionnelle's ethics committee. CGN cultures were prepared from 7-day-old murine pups (C57BL/6J mice, Charles River Laboratories) as described by Miller and Johnson [31] with slight modifications. Briefly, freshly dissected cerebella were incubated for 10 min at 37°C with 0.25 mg/ml trypsin and cells were dissociated in HBSS -Ca2+ -Mg2+ in the presence of 0.5 mg/ml trypsin inhibitor and 0.1 mg/ml DNaseI by several steps of mechanical disruption. The resulting cell suspension was centrifuged and resuspended in K25+S medium (Basal Medium Eagle [BME] supplemented with 10% fetal bovine serum, 2 mM L-glutamine, 10 mM HEPES, penicillin-streptomycin 100 IU/ml-100 μg/ml and 20 mM KCl to achieve a final concentration of 25 mM). The cell suspension was filtered through a 40 μm cell strainer (Falcon) and plated in a coated dish for 25 min to allow attachment of non-neuronal cells. Neurons were then resuspended, counted and seeded at a density of 25.10^4 ^cells/cm^2 ^in culture dishes coated with poly-D-lysine (Becton Dickinson Biosciences). The granule neurons were cultured at 37°C in a humidified incubator with 6% CO_2 _/94% air for 7 days. To prevent proliferation of remaining non-neuronal cells, 10 μM cytosine β-D-arabinofuranoside (Ara-C) was added to the culture medium 24 h after plating. At 7 days *in vitro*, granule neurons represented more than 98% of cultured cells (data not shown).

CGN were washed and incubated for the indicated times in serum-free BME supplemented with L-Gln, HEPES, antibiotics and 1 μM of the NMDA antagonist (+)-MK-801, and containing either 25 mM KCl (K25 medium) or 5 mM KCl (K5 medium). We chose to use K25 medium as a control instead of initial culture medium to exclude gene expression differences resulting from serum deprivation, which has been shown to induce the death of a small proportion of cultured CGN [31]. Moreover, we added 1 μM of MK-801 in both K25 and K5 media to avoid any change in gene expression due to non-controlled NMDA receptor stimulation by endogenously released glutamate.

### RNA preparation, reverse transcription and real-time PCR

Total RNA was prepared using Trizol (Invitrogen) according to the manufacturer's instruction. Purified RNA was treated with the DNase I from the DNA-free™ kit (Ambion) according to manufacturer's instructions. For reverse transcription, 2 μg of total RNA were reverse transcribed using 200 U M-MLV reverse transcriptase (Invitrogen) in the presence of 2.5 μM random hexamers and 0.5 mM dNTP. Three independent RT reactions were performed from each RNA preparation. Four ng of the resulting cDNAs were used as template for real time PCR using ABI Prism 7000 with the SybrGreen^® ^PCR Master Mix (Applied Biosystems). Primers were designed with Primer Express™ software (Applied Biosystems). The sequences of all the primers used were deposited in the Gene Expression Omnibus (GEO) repository under the accession number GPL7701. The PCR reaction was performed in 10 μl in the presence of 300 nM specific primers. Thermal cycling parameters were 2 min at 50 °C, 10 min at 95°C, followed by 40 cycles of 95°C for 15 s and 60°C for 1 min. Data were analyzed with ABI prism 7000 SDS software. The level of expression of each GPCR "X" was normalized to the geometric mean of the expression levels of the selected reference genes, R1 to R3, in the same PCR plate according to the formula:

Reference genes were selected according to the GeNorm procedure [18]. Reference genes used in this study were *B2m *(beta-2 microglobulin), *Gapdh *(glyceraldehyde-3-phosphate dehydrogenase), and *Gusb *(glucuronidase, beta).

### Statistical analysis of PCR data

To reduce the variability introduced by reverse transcription (RT), the normalized, log2-transformed PCR data obtained from individual RT were mean-centered and scaled in SD units. Statistical tests were performed with the statistical module of MeV 4.2 [17]. For Kruskal-Wallis test, post-hoc tests were performed with XLStat (Addinsoft). All p-value were corrected for multiple testing according to the method proposed by Hochberg and Benjamini [16].

## List of abbreviations used

GPCR: G protein-coupled receptor; CGN: cerebellar granule neuron; DIV: day(s) *in vitro*; DE: differentially expressed; ORF: open reading frame; 7TM: seven transmembrane.

## Competing interests

The authors declare that they have no competing interests.

## Authors' contributions

BM carried out the cerebellar granule neuron cultures, performed the real-time PCR experiments for selecting statistical tests, and helped to draft the manuscript. ALD validated the primer collection, and performed the real-time PCR experiments on cerebellar granule neurons. CD helped with the management of real-time PCR data and statistics. LJ designed the study, identified the endo-GPCR repertoire, processed real-time PCR data, performed statistical analysis, and drafted the manuscript. All authors read and approved the final manuscript.

## Supplementary Material

Additional file 1 **List of the GPCRs studied in this manuscript**. An Excel file listing the names of the GPCRs, as well as aliases, Unigene, Entrez and Ensembl gene ID. Click here for file

Additional file 2 **Comparison of Taqman^®^- and SybrGreen^®^-based detection assays**. An Excel file that lists the results of the assays performed to compare commercial Taqman^® ^probes to the SybrGreen^®^-based detection system used in this study. Click here for file

Additional file 3 **Sequences of the primers used in this manuscript**. An Excel file that gives the sequences of the primers used for each GPCR in this study. Click here for file

Additional file 4 **Results of the replicated PCR experiments to test for normality of Ct distribution**. An Excel file that lists the 9 GPCRs that were used for replicated PCR experiments on genomic DNA as well as the results of the statistical analysis conducted to determine if Ct distributions displayed on Additional files 5, 6, 7, 8, 9, 10, 11, 12 and 13 are Gaussian Click here for file

Additional file 5 **Distribution of the Ct values of replicated PCR experiment using *Avpr2 *primers**. A pdf file showing the distribution of the Ct values obtained in 64-replicate RT-PCR experiments with *Avpr2 *primers. Click here for file

Additional file 6 **Distribution of the Ct values of replicated PCR experiment using *Adrb2 *primers**. A pdf file showing the distribution of the Ct values obtained in 64-replicate RT-PCR experiments with *Adrb2 *primers. Click here for file

Additional file 7 **Distribution of the Ct values of replicated PCR experiment using *Npy2r *primers**. A pdf file showing the distribution of the Ct values obtained in 64-replicate RT-PCR experiments with *Npy2r *primers. Click here for file

Additional file 8 **Distribution of the Ct values of replicated PCR experiment using *Chrm1 *primers**. A pdf file showing the distribution of the Ct values obtained in 64-replicate RT-PCR experiments with *Chrm1 *primers. Click here for file

Additional file 9 **Distribution of the Ct values of replicated PCR experiment using *Htr2c *primers**. A pdf file showing the distribution of the Ct values obtained in 64-replicate RT-PCR experiments with *Htr2c *primers. Click here for file

Additional file 10 **Distribution of the Ct values of replicated PCR experiment using *Gcgr *primers**. A pdf file showing the distribution of the Ct values obtained in 64-replicate RT-PCR experiments with *Gcgr *primers. Click here for file

Additional file 11 **Distribution of the Ct values of replicated PCR experiment using *Adcyap1r1 *primers**. A pdf file showing the distribution of the Ct values obtained in 64-replicate RT-PCR experiments with *Adcyap1r1 *primers. Click here for file

Additional file 12 **Distribution of the Ct values of replicated PCR experiment using *Casr *primers**. A pdf file showing the distribution of the Ct values obtained in 64-replicate RT-PCR experiments with *Casr *primers. Click here for file

Additional file 13 **Distribution of the Ct values of replicated PCR experiment using *Fzd4 *primers**. A pdf file showing the distribution of the Ct values obtained in 64-replicate RT-PCR experiments with *Fzd4 *primers. Click here for file

Additional file 14 **List of the GPCRs differentially expressed in CGNs incubated in 5 (K5) or 25 (K25) mM KCl for 4 hours**. An Excel file that lists the names of those GPCRs differentially expressed, expression values and fold-change. Click here for file

Additional file 15 **List of GPCRs differentially expressed during the differentiation of CGNs *in vitro***. An Excel file that lists the names of those GPCRs differentially expressed in at least one condition during the developmental time course, normalized expression values, average and standard deviation Click here for file
