# Supplementary figures and images for: Genome-wide profiling of G protein-coupled receptors in cerebellar granule neurons using high-throughput, real-time PCR
Source: BMC Genomics. 2011 May 16;12:241. doi: 10.1186/1471-2164-12-241 (PMC3111393; doi:10.1186/1471-2164-12-241)

## Avpr2

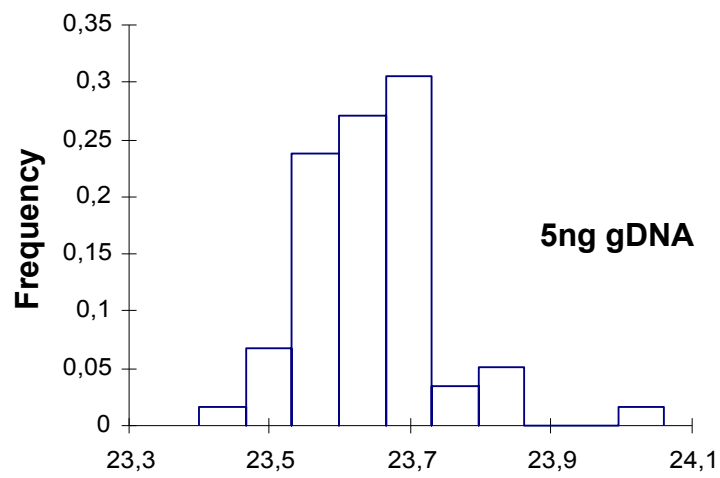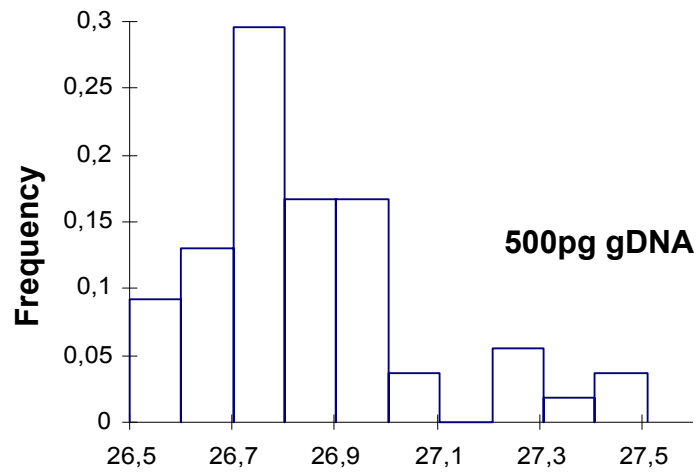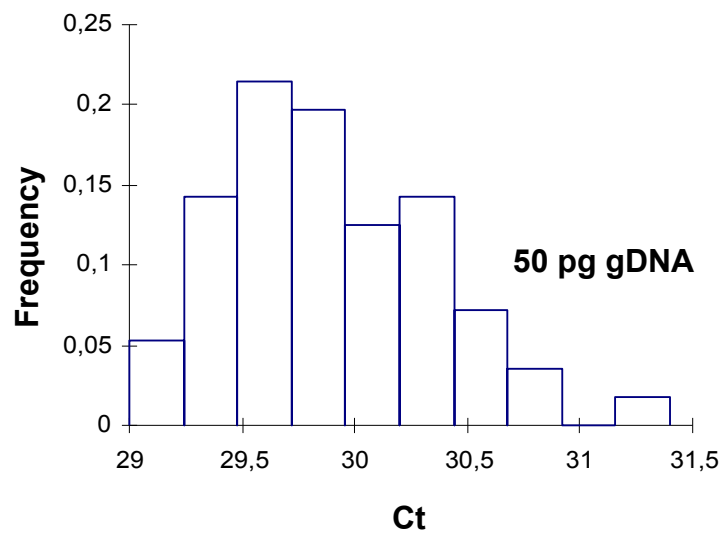

Supplement: Additional file 5 — Distribution of the Ct values of replicated PCR experiment using Avpr2 primers. A pdf file showing the distribution of the Ct values obtained in 64-replicate RT-PCR experiments with Avpr2 primers. [file 1471-2164-12-241-S5.PDF]

## Adrb2

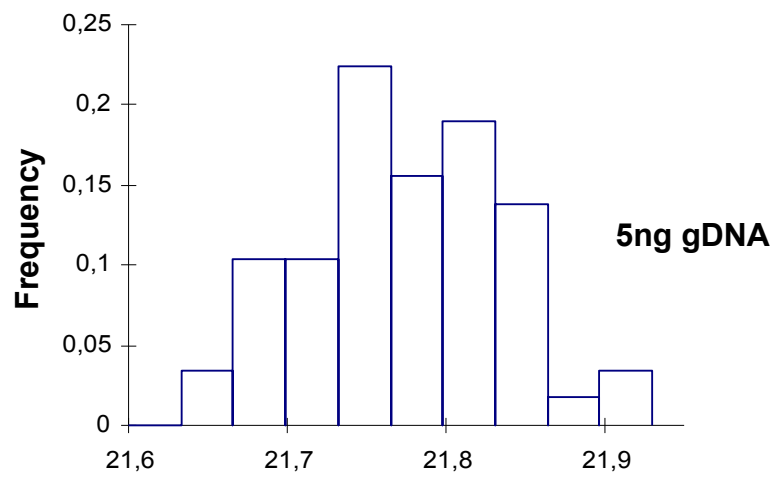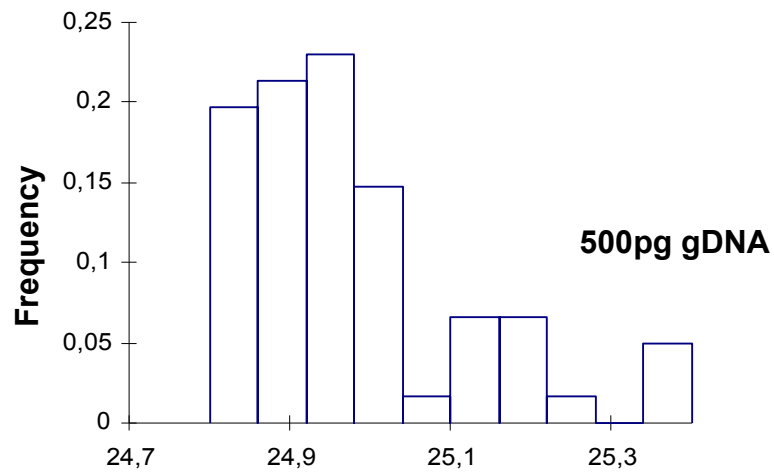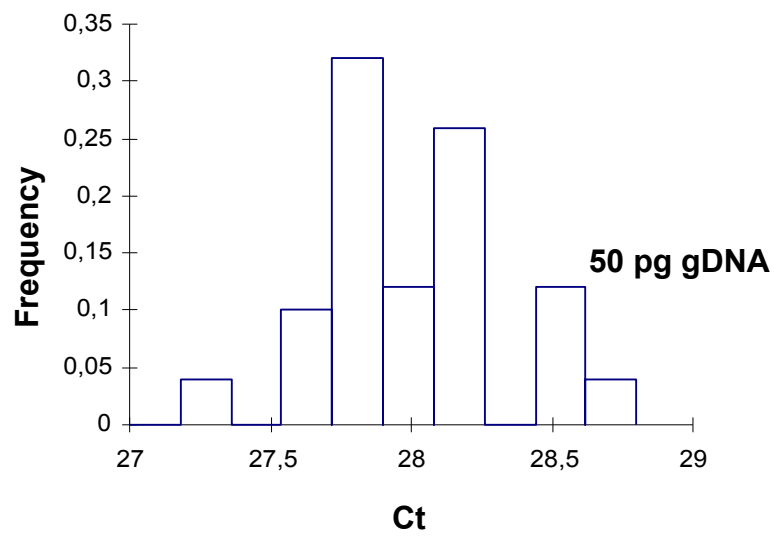

Supplement: Additional file 6 — Distribution of the Ct values of replicated PCR experiment using Adrb2 primers. A pdf file showing the distribution of the Ct values obtained in 64-replicate RT-PCR experiments with Adrb2 primers. [file 1471-2164-12-241-S6.PDF]

# Npy2r

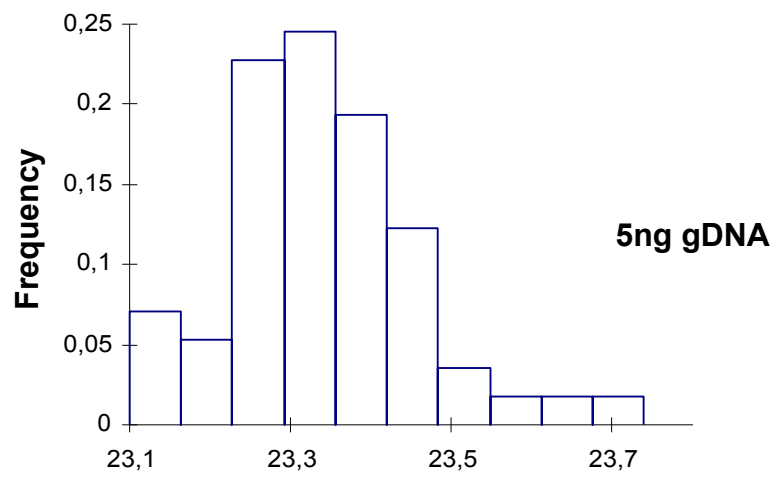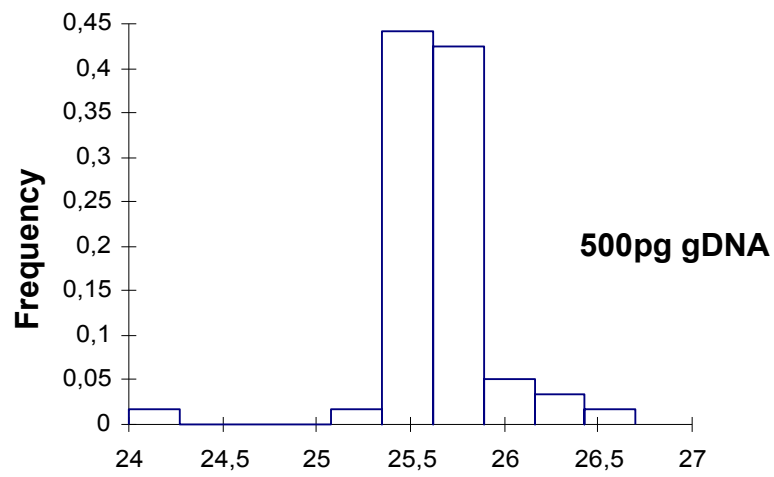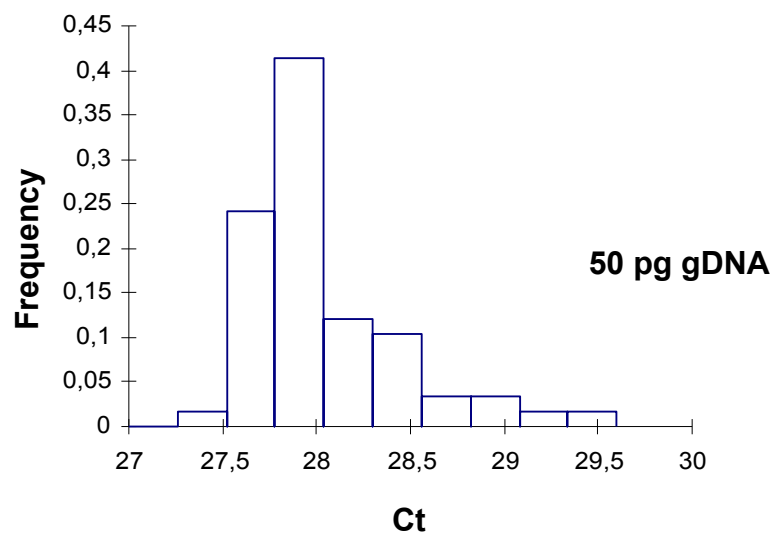

Supplement: Additional file 7 — Distribution of the Ct values of replicated PCR experiment using Npy2r primers. A pdf file showing the distribution of the Ct values obtained in 64-replicate RT-PCR experiments with Npy2r primers. [file 1471-2164-12-241-S7.PDF]

# Chrm1

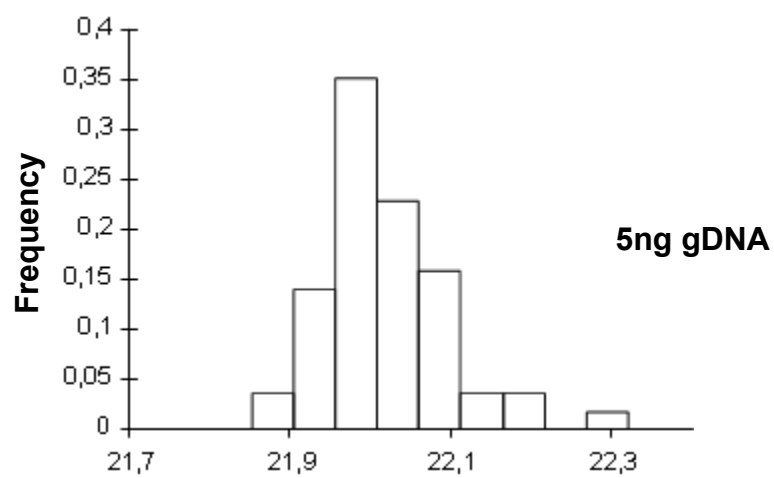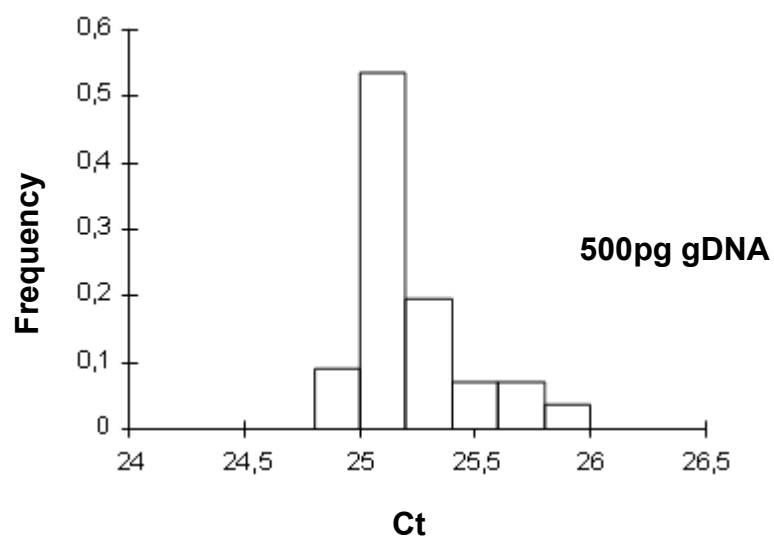

Supplement: Additional file 8 — Distribution of the Ct values of replicated PCR experiment using Chrm1 primers. A pdf file showing the distribution of the Ct values obtained in 64-replicate RT-PCR experiments with Chrm1 primers. [file 1471-2164-12-241-S8.PDF]

## Htr2c

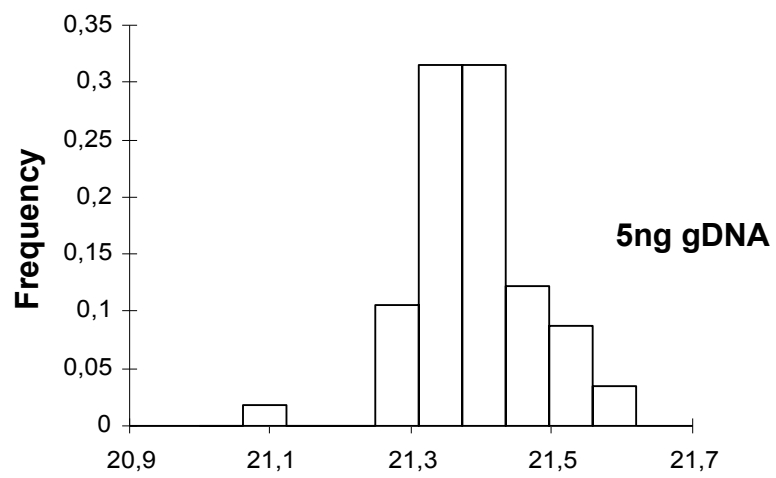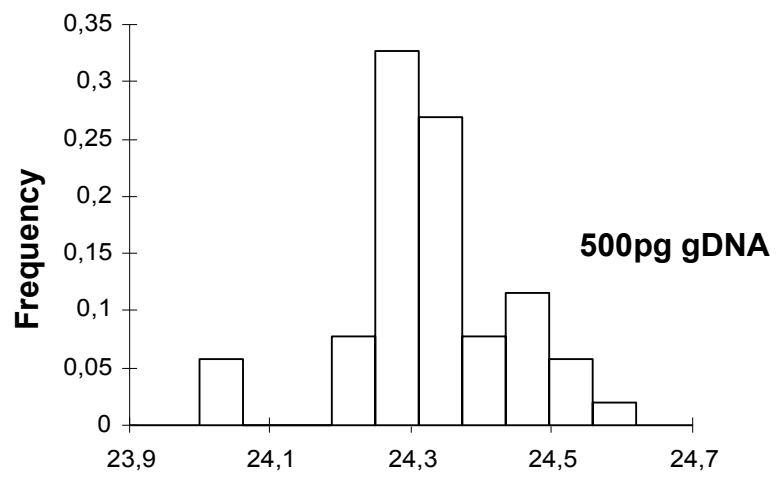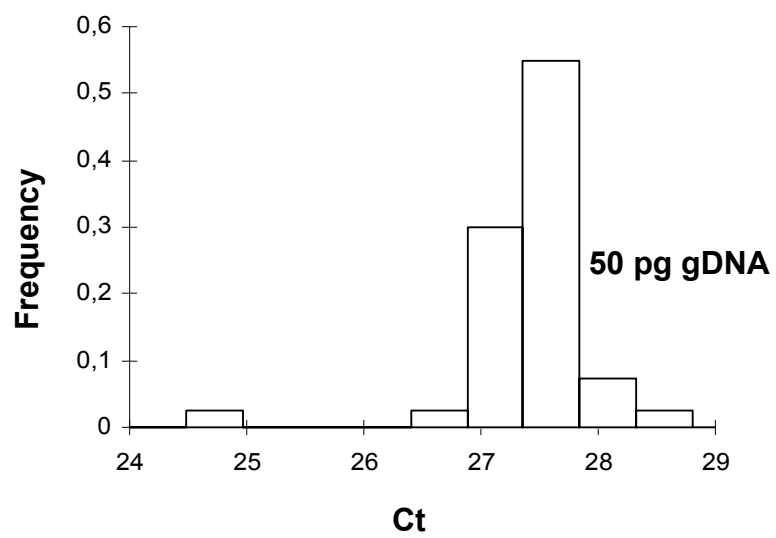

Supplement: Additional file 9 — Distribution of the Ct values of replicated PCR experiment using Htr2c primers. A pdf file showing the distribution of the Ct values obtained in 64-replicate RT-PCR experiments with Htr2c primers. [file 1471-2164-12-241-S9.PDF]

Gcgr

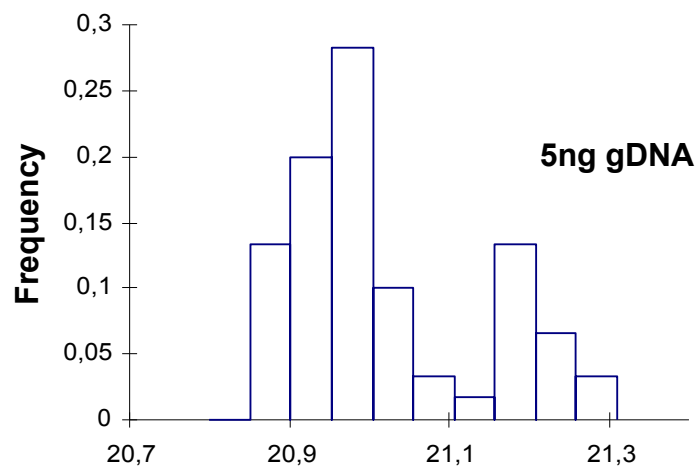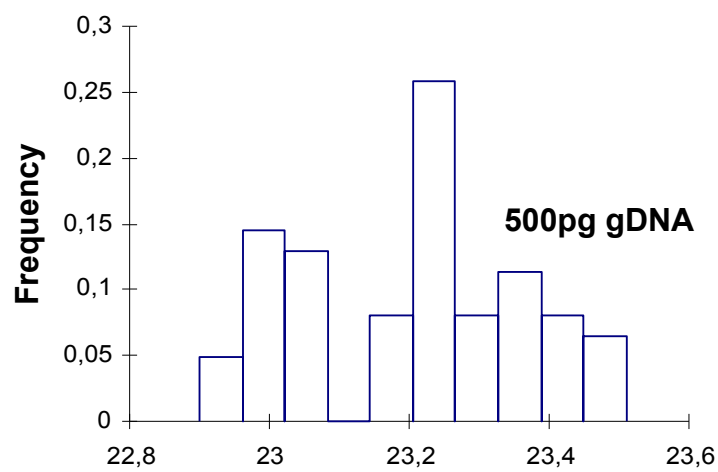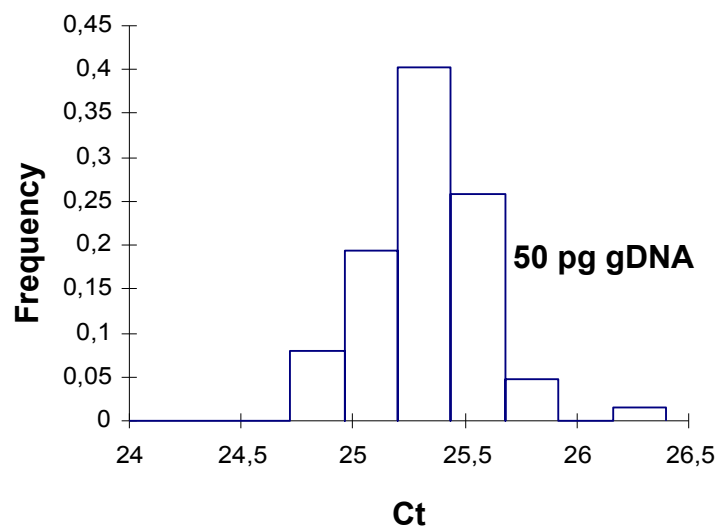

Supplement: Additional file 10 — Distribution of the Ct values of replicated PCR experiment using Gcgr primers. A pdf file showing the distribution of the Ct values obtained in 64-replicate RT-PCR experiments with Gcgr primers. [file 1471-2164-12-241-S10.PDF]

## Adcyap1r1

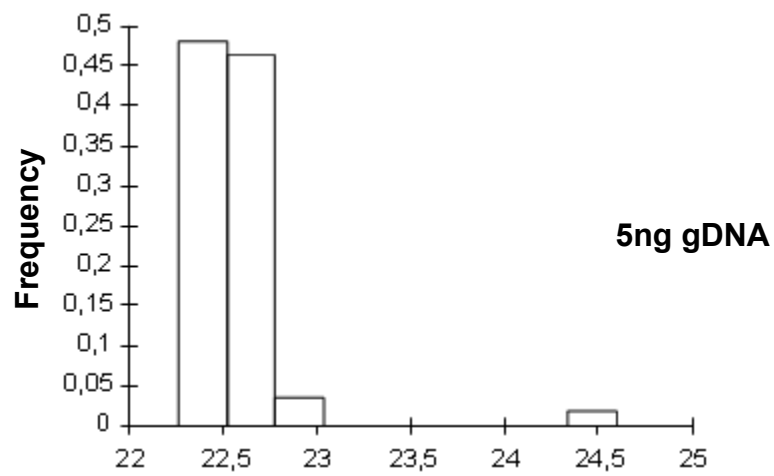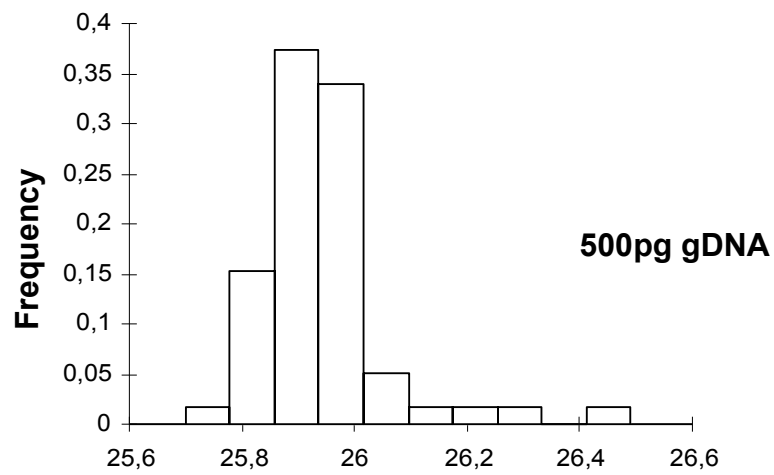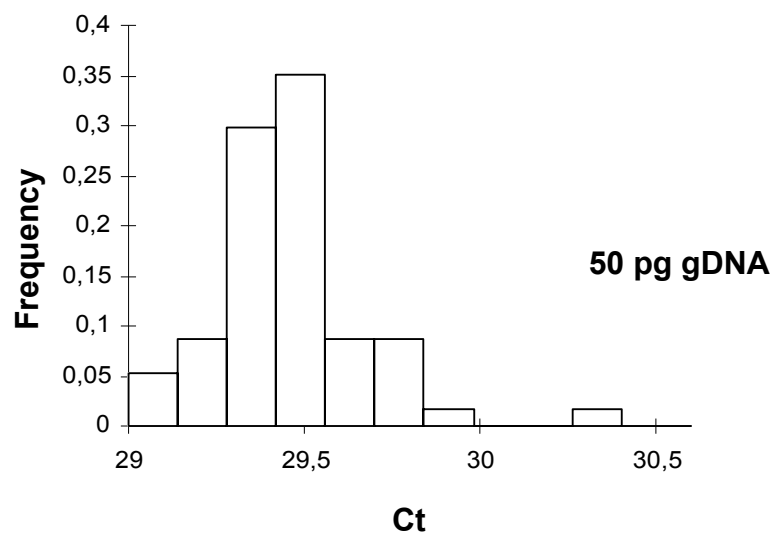

Supplement: Additional file 11 — Distribution of the Ct values of replicated PCR experiment using Adcyap1r1 primers. A pdf file showing the distribution of the Ct values obtained in 64-replicate RT-PCR experiments with Adcyap1r1 primers. [file 1471-2164-12-241-S11.PDF]

Casr

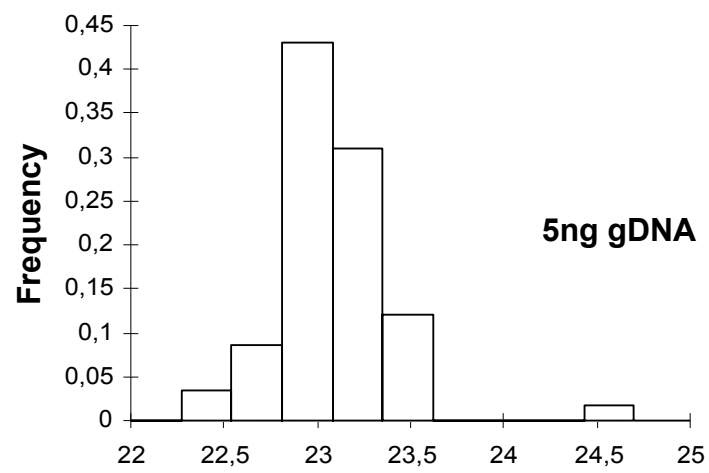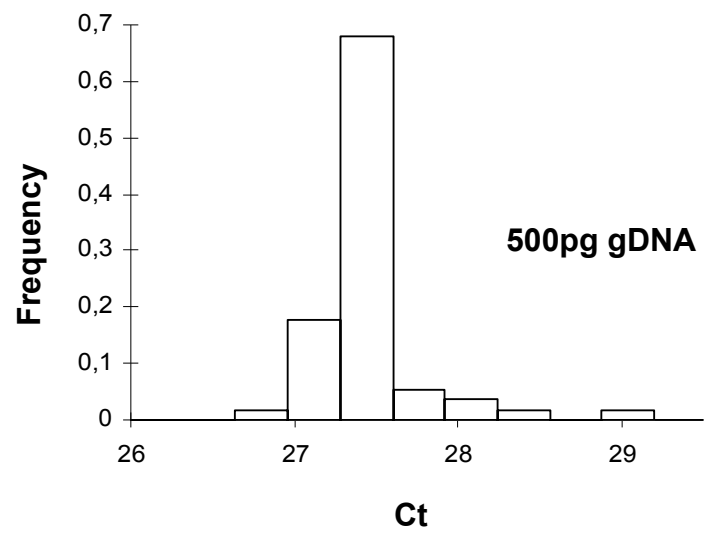

Supplement: Additional file 12 — Distribution of the Ct values of replicated PCR experiment using Casr primers. A pdf file showing the distribution of the Ct values obtained in 64-replicate RT-PCR experiments with Casr primers. [file 1471-2164-12-241-S12.PDF]

# Fzd4

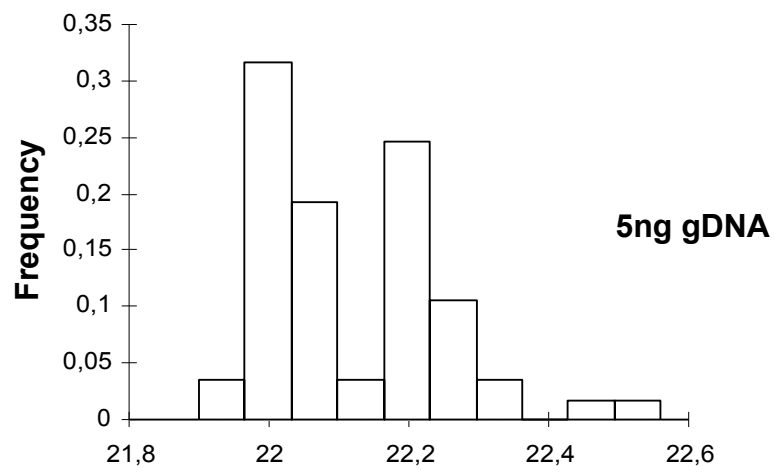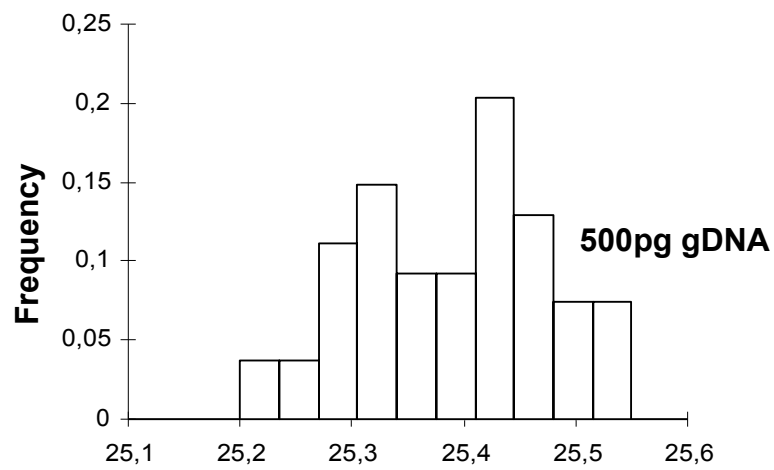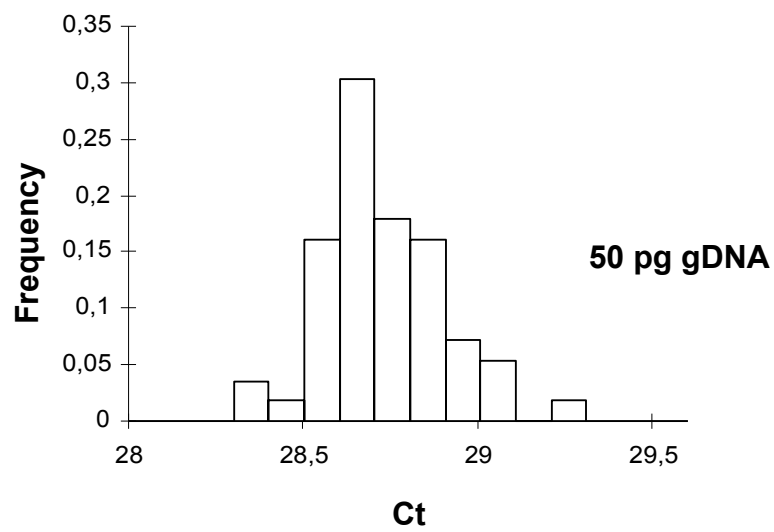

Supplement: Additional file 13 — Distribution of the Ct values of replicated PCR experiment using Fzd4 primers. A pdf file showing the distribution of the Ct values obtained in 64-replicate RT-PCR experiments with Fzd4 primers. [file 1471-2164-12-241-S13.PDF]
